# Supplementary material for: Associations of timing of physical activity with all-cause and cause-specific mortality in a prospective cohort study
Source: Nat Commun. 2023 Feb 18;14:930. doi: 10.1038/s41467-023-36546-5 (PMC9938683; doi:10.1038/s41467-023-36546-5)
Supplement: Supplementary file 1 — Supplementary Information [file 41467_2023_36546_MOESM1_ESM.pdf]

Supplementary material

Supplementary Fig. 1. Standardized risk of all-cause mortality according to MVPA timing groups 3

Supplementary Fig. 2. Standardized risk of CVD mortality according to MVPA timing groups 4

Supplementary Fig. 3. Standardized risk of cancer mortality according to MVPA timing groups 5

Supplementary Fig. 4. Flowchart of participant enrolment 6

Supplementary Fig. 5. The exploratory analysis on the associations between timing of MVPA and mortality risk 7

Supplementary Fig. 6. Timeline of some covariates collection 8

Supplementary Table 1. Sensitivity analysis on the associations between timing of MVPA and mortality risk by using competing risk regression (Fine and Gray) 9

Supplementary Table 2. Sensitivity analysis on the associations between timing of MVPA and mortality risk by using the dataset without imputation 10

Supplementary Table 3. Sensitivity analysis on the associations between timing of MVPA and mortality risk by excluding participants with shift work history 11

Supplementary Table 4. Sensitivity analysis on the associations between timing of MVPA and mortality risk by excluding participants who wore accelerometers during the daylight saving time transition 12

Supplementary Table 5. Sensitivity analysis on the associations between timing of MVPA and mortality risk by controlling for month of accelerometer wear 13

Supplementary Table 6. Sensitivity analysis on the associations between timing of MVPA and mortality risk by additionally adjusting for health-related variables potentially on the causal pathway 14

Supplementary Table 7. Sensitivity analysis on the associations between timing of MVPA and mortality risk by excluding events within the first year of follow-up 15

Supplementary Table 8. Sensitivity analysis on the associations between timing of MVPA and mortality risk by censoring up to Dec 31, 2019 16

Supplementary Table 9. Sensitivity analysis on the associations between timing of MVPA and mortality risk by using the subsample with ≥6 days of accelerometer wear 17

Supplementary Table 10. Interaction effects of timing of MVPA and age categories (< 65 and ≥ 65 years) on mortality risk 18

Supplementary Table 11. Interaction effects of timing of MVPA and sex on mortality risk 19

Supplementary Table 12. Interaction effects of timing of MVPA and MVPA level on mortality risk 20

Supplementary Table 13. Interaction effects of timing of MVPA and CVDs on mortality risk 21

Supplementary Table 14. Interaction effects of timing of MVPA and obesity on mortality risk 22

Supplementary Table 15. Subgroup analysis on the associations between timing of MVPA and mortality risk stratified by age categories (< 65 and ≥ 65 years) 23

Supplementary Table 16. Subgroup analysis on the associations between timing of MVPA and mortality risk stratified by sex 24

Supplementary Table 17. Subgroup analysis on the associations between timing of MVPA and mortality risk stratified by MVPA levels 25

Supplementary Table 18. Subgroup analysis on the associations between timing of MVPA and mortality risk stratified by CVDs 26

Supplementary Table 19. Subgroup analysis on the associations between timing of MVPA and mortality risk stratified by obesity 27

Supplementary Table 20. The information sources of exposure, outcomes, and covariates 28

Supplementary Table 21. The numbers (percentages) of participants with missing covariates 29

Supplementary Table 22. Baseline characteristics of overall sample and complete case sample 30

Supplementary Fig. 1. Standardized risk of all-cause mortality according to MVPA timing groups

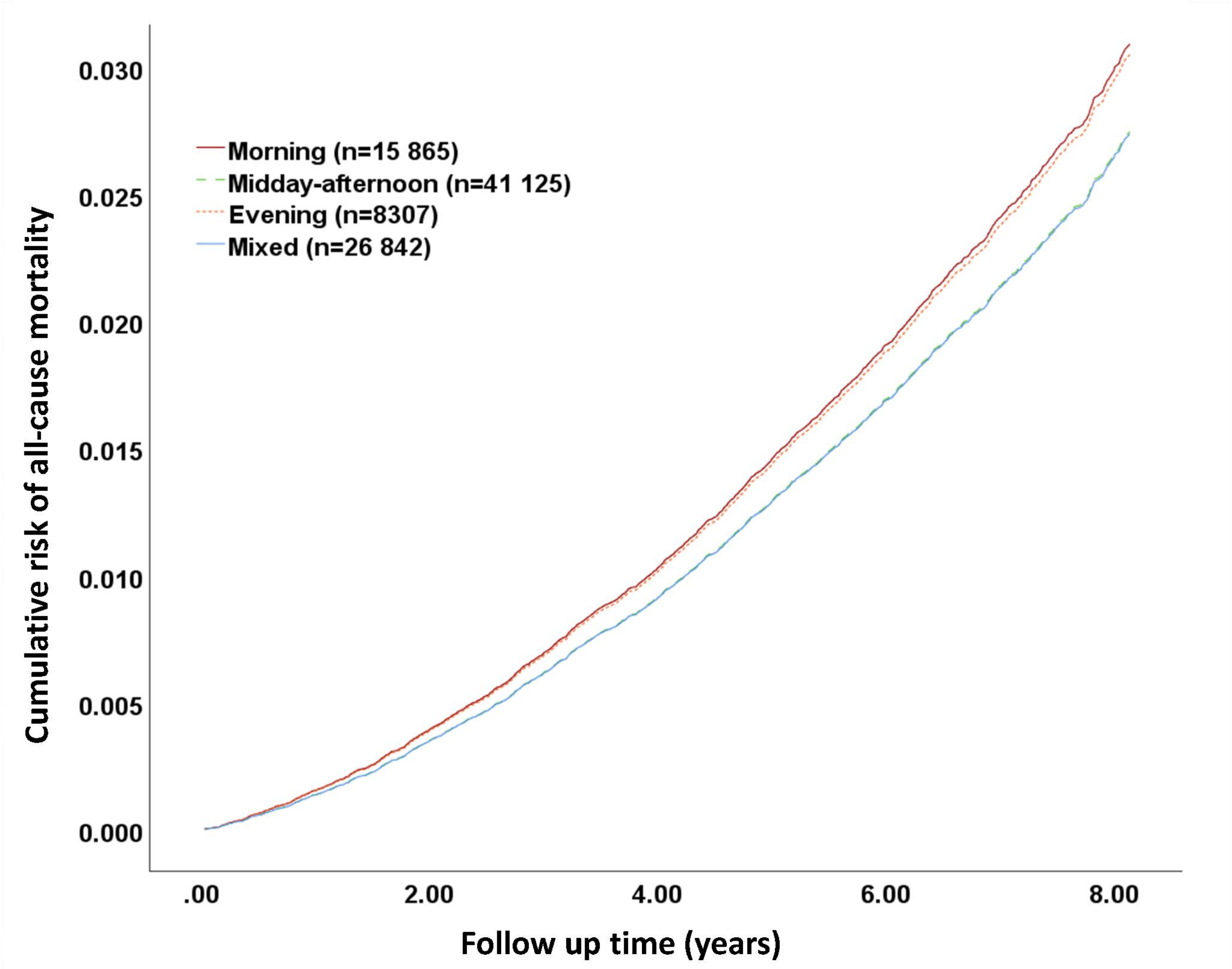

MVPA: moderate to vigorous physical activity. Analyses were adjusted for age and sex, ethnicity, Townsend index of deprivation, recruitment center, education level, season of accelerometer wear, healthy diet score, smoking status, alcohol intake, sleep duration (< 7 hours, 7-8 hours, > 8 hours), sleep midpoint, and total MVPA volumes.

Supplementary Fig. 2. Standardized risk of CVD mortality according to MVPA timing groups

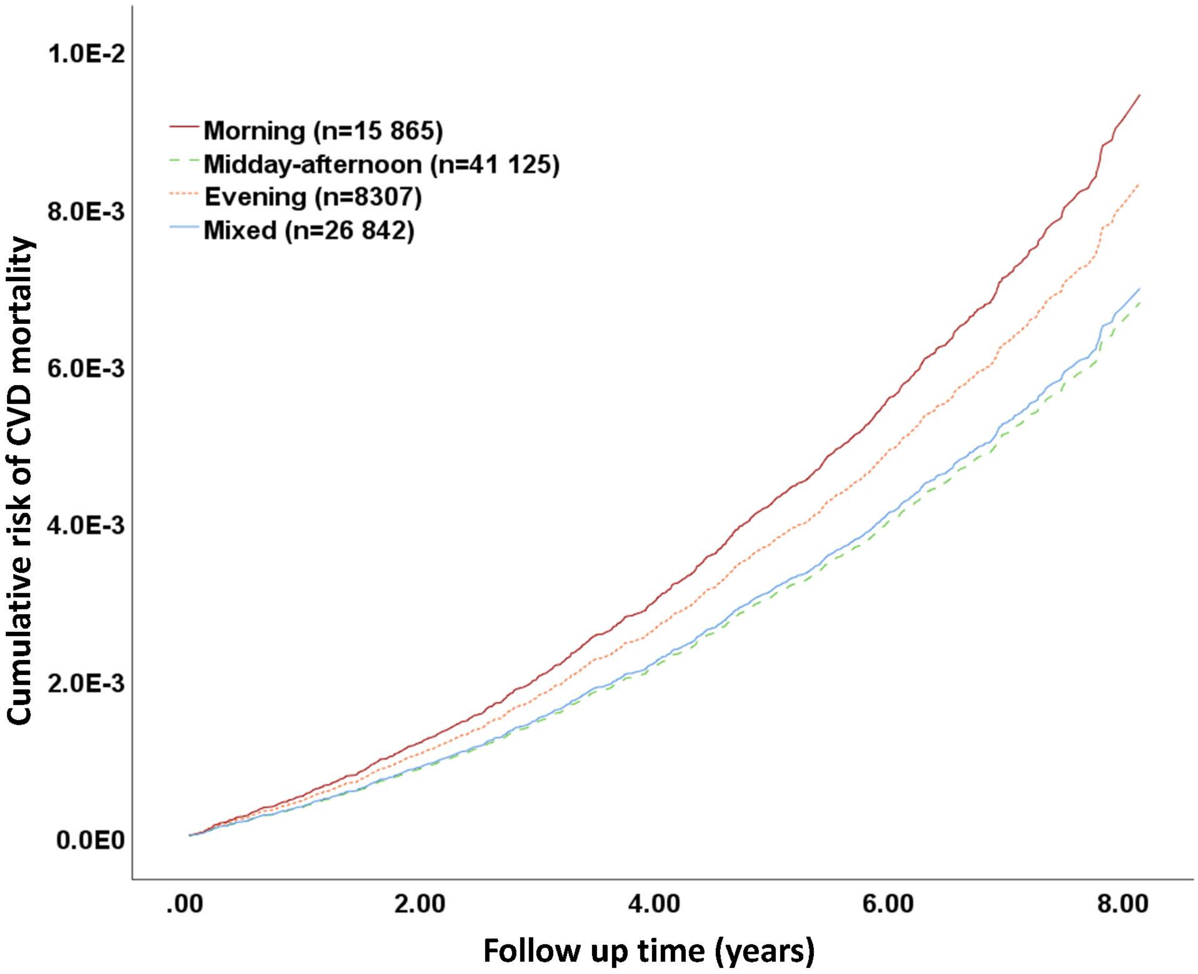

CVD: cardiovascular disease; **MVPA**: moderate to vigorous physical activity. Analyses were adjusted for age and sex, ethnicity, Townsend index of deprivation, recruitment center, education level, season of accelerometer wear, healthy diet score, smoking status, alcohol intake, sleep duration (< 7 hours, 7-8 hours, > 8 hours), sleep midpoint, and total MVPA volumes.

Supplementary Fig. 3. Standardized risk of cancer mortality according to MVPA timing groups

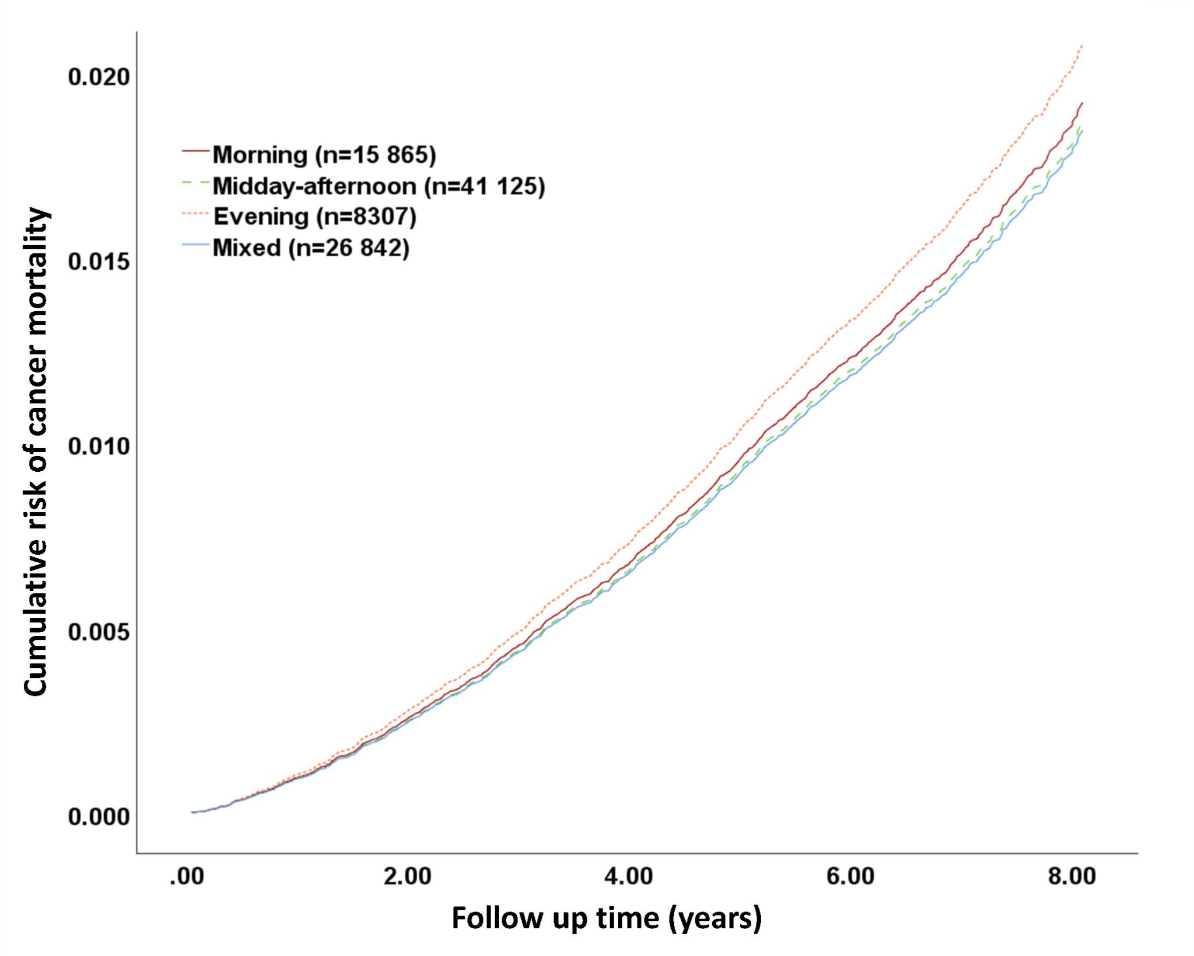

MVPA: moderate to vigorous physical activity. Analyses were adjusted for age and sex, ethnicity, Townsend index of deprivation, recruitment center, education level, season of accelerometer wear, healthy diet score, smoking status, alcohol intake, sleep duration (< 7 hours, 7-8 hours, > 8 hours), sleep midpoint, and total MVPA volumes.

Supplementary Fig. 4. Flowchart of participant enrolment

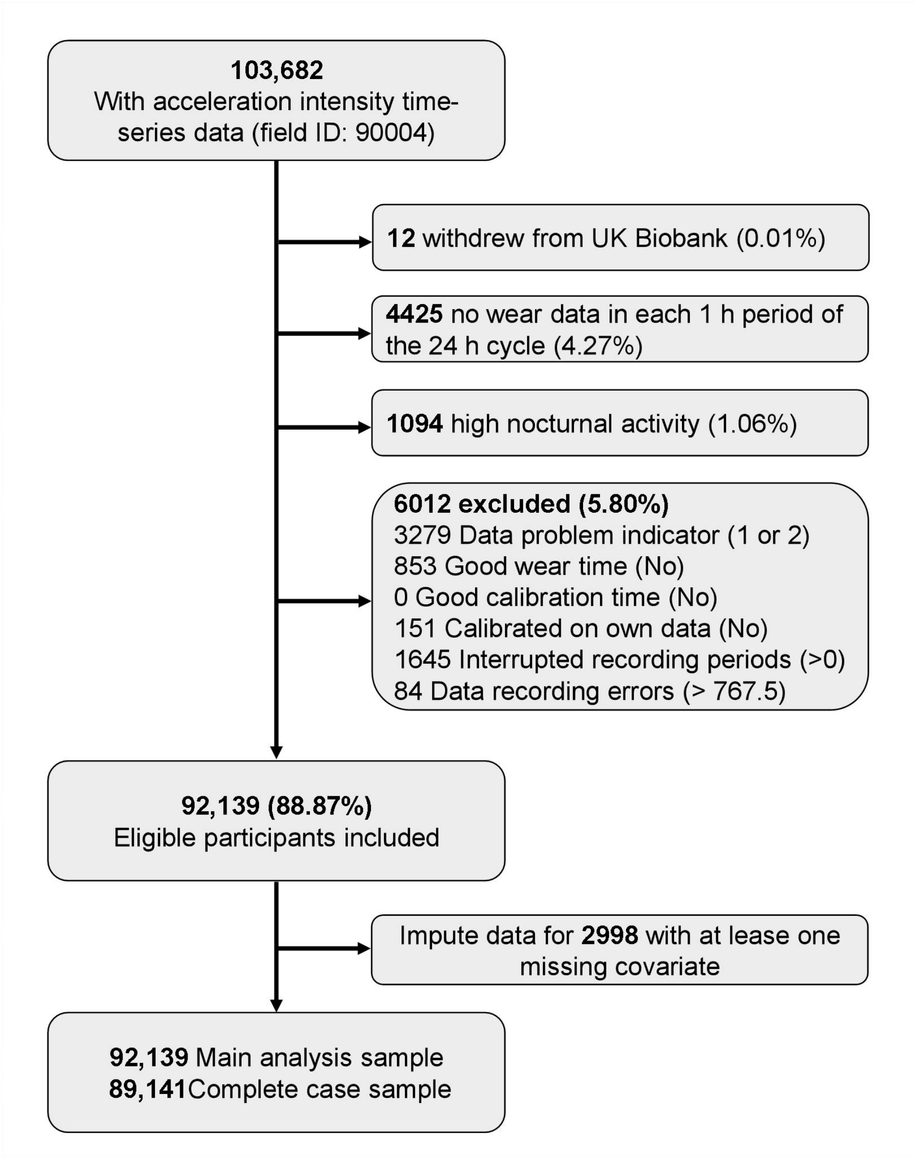

Supplementary Fig. 5. The exploratory analysis on the associations between timing of MVPA and mortality risk

Outcomes	No. of events	Total No. of participants	Hazards Ratio (95%CI)
**All cause mortality**			
Mixed	2274	74,288	1.00 (reference)
05:00-07:00	11	250	1.46 (0.80-2.64)
07:00-09:00	105	2022	1.49 (1.22-1.81)
09:00-11:00	238	4694	1.19 (1.04-1.36)
11:00-13:00	165	3830	0.99 (0.84-1.16)
13:00-15:00	121	2562	1.12 (0.93-1.34)
15:00-17:00	86	2127	0.98 (0.80-1.22)
17:00-19:00	41	1210	1.17 (0.86-1.60)
19:00-21:00	26	820	1.11 (0.76-1.64)
21:00-24:00	21	336	1.51 (0.98-2.32)
**CVD mortality**			
Mixed	760	74,288	1.00 (reference)
05:00-07:00	6	250	2.02 (0.90-4.54)
07:00-09:00	42	2022	1.66 (1.21-2.26)
09:00-11:00	101	4694	1.42 (1.15-1.75)
11:00-13:00	56	3830	0.94 (0.72-1.24)
13:00-15:00	41	2562	1.06 (0.77-1.45)
15:00-17:00	36	2127	1.16 (0.83-1.62)
17:00-19:00	17	1210	1.40 (0.86-2.26)
19:00-21:00	10	820	1.25 (0.67-2.33)
21:00-24:00	7	336	1.36 (0.64-2.86)
**Cancer mortality**			
Mixed	1396	74,288	1.00 (reference)
05:00-07:00	6	250	1.39 (0.62-3.11)
07:00-09:00	52	2022	1.24 (0.94-1.63)
09:00-11:00	138	4694	1.16 (0.97-1.38)
11:00-13:00	97	3830	0.99 (0.80-1.21)
13:00-15:00	72	2562	1.13 (0.89-1.44)
15:00-17:00	54	2127	1.04 (0.79-1.36)
17:00-19:00	27	1210	1.28 (0.87-1.87)
19:00-21:00	18	820	1.28 (0.80-2.04)
21:00-24:00	12	336	1.51 (0.85-2.67)

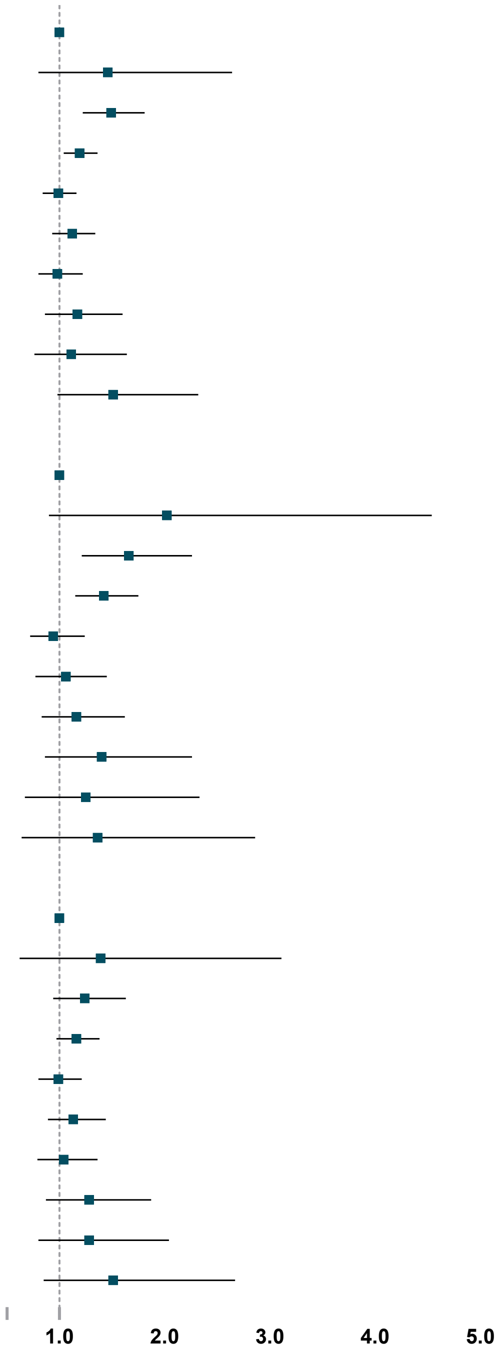

[a] All *P* values remained significant after multiple testing with FDR method. **CVDs**: cardiovascular diseases. Cox proportional hazard regression was used to examine the associations, which were adjusted for age, sex, ethnicity, Townsend index of deprivation, recruitment center, education level, season of accelerometer wear, healthy diet score, smoking status, alcohol intake, sleep duration (< 7 hours, 7-8 hours, > 8 hours), sleep midpoint, and total MVPA volume (**Model 3**). **CVD**: cardiovascular disease; **HR**: hazard ratio; **MVPA**: moderate to vigorous physical activity. Error bars represent the 95% confidence intervals for each effect estimate.

Supplementary Fig. 6. Timeline of some covariates collection

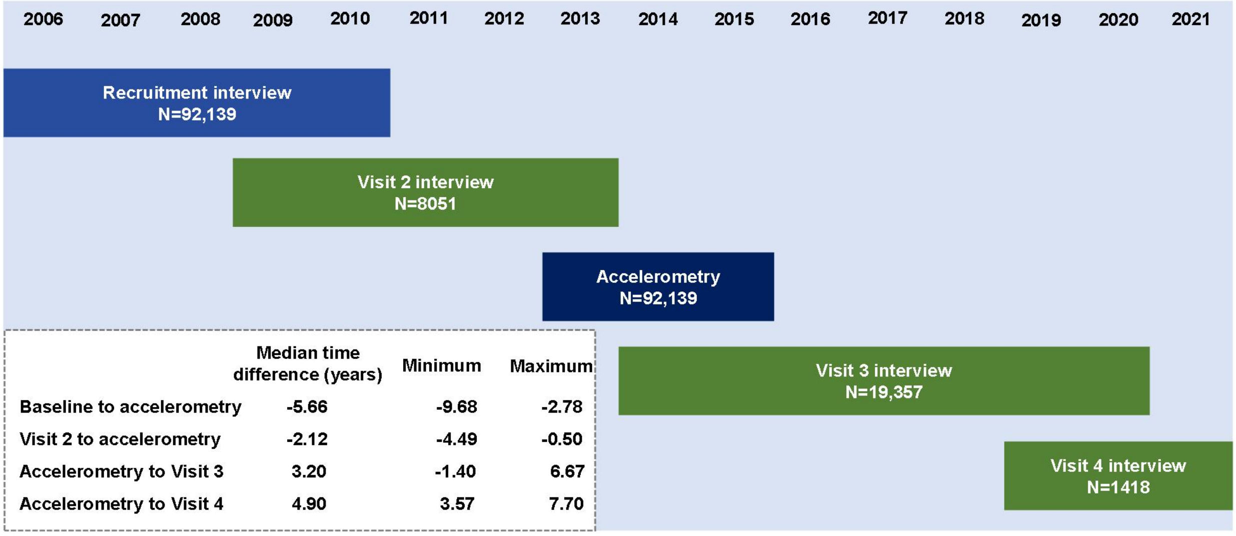

	Median time difference (years)	Minimum	Maximum
Baseline to accelerometry	-5.66	-9.68	-2.78
Visit 2 to accelerometry	-2.12	-4.49	-0.50
Accelerometry to Visit 3	3.20	-1.40	6.67
Accelerometry to Visit 4	4.90	3.57	7.70

The covariates with repeated measurements include education level, smoking status, alcohol consumption, healthy diet score, obesity, diabetes history, longstanding illness, and cancer history were obtained from touchscreen questionnaires at the time-point closest to the accelerometry.

Supplementary Table 1. Sensitivity analysis on the associations between timing of MVPA and mortality risk by using competing risk regression (Fine and Gray)

Outcomes	Events	n	Person-years	Model 1 HR (95% CI); *P*	Model 2 HR (95% CI); *P*	Model 3 HR (95% CI); *P*[a]
**CVD mortality**	1076	92,139	638,825			
Morning	274	15,865	109,537	1.00 (reference)	1.00 (reference)	1.00 (reference)
Midday-afternoon	464	41,125	284,481	0.66 (0.57-0.77); 4.8e-8	0.68 (0.59-0.79); 6.8e-7	0.72 (0.62-0.84); 2.0e-5[a]
Evening	90	8307	57,768	0.94 (0.74-1.20); 0.62	0.95 (0.75-1.21); 0.67	0.86 (0.68-1.10); 0.23
Mixed	248	26,842	187,039	0.72 (0.60-0.85); 1.8e-4	0.73 (0.62-0.87); 4.7e-4	0.74 (0.62-0.88); 7.6e-4[a]
**Cancer mortality**	1872	92,139	638,825			
Morning	362	15,865	109,537	1.00 (reference)	1.00 (reference)	1.00 (reference)
Midday-afternoon	888	41,125	284,481	0.96 (0.85-1.08); 0.48	0.97 (0.86-1.10); 0.64	0.98 (0.86-1.11); 0.70
Evening	154	8307	57,768	1.14 (0.94-1.38); 0.19	1.14 (0.94-1.38); 0.18	1.08 (0.89-1.31); 0.46
Mixed	468	26,842	187,039	0.96 (0.84-1.10); 0.56	0.97 (0.84-1.11); 0.65	0.97 (0.84-1.11); 0.65

[a] All *P* values remained significant after multiple testing with the FDR method. Competing risk regression was used to examine the associations. **Model 1** was adjusted for age and sex. **Model 2** was adjusted as in model 1 and for ethnicity, Townsend index of deprivation, recruitment center, education level, season of accelerometer wear, smoking status, and alcohol intake, healthy diet score. **Model 3** was adjusted as in model 2 and for sleep duration (< 7 hours, 7-8 hours, > 8 hours), sleep midpoint, and total MVPA volume. **CVD**: cardiovascular disease; **HR**: hazard ratio; **MVPA**: moderate to vigorous physical activity. In the competing risk regression models [Gray, Ann Stat. 1988], the Fine-Gray subdistribution hazards were calculated, incorporating other-cause death as a competing risk for cause-specific mortality.

Supplementary Table 2. Sensitivity analysis on the associations between timing of MVPA and mortality risk by using the dataset without imputation

Outcomes	Events	n	Person-years	Model 1 HR (95% CI); *P*	Model 2 HR (95% CI); *P*	Model 3 HR (95% CI); *P*[a]
**All-cause mortality**	2950	89,141	618,004			
Morning	616	15,312	105,750	1.00 (reference)	1.00 (reference)	1.00 (reference)
Midday-afternoon	1373	39,763	274,999	0.86 (0.78-0.95); 0.002	0.88 (0.80-0.97); 0.009	0.90 (0.81-0.99); 0.03
Evening	238	8009	55,706	1.06 (0.91-1.23); 0.44	1.06 (0.92-1.24); 0.42	0.99 (0.85-1.15); 0.85
Mixed	723	26,057	181,549	0.88 (0.79-0.98); 0.02	0.89 (0.80-0.99); 0.04	0.89 (0.80-0.99); 0.04
**CVD mortality**	1027	89,141	618,004			
Morning	260	15,312	105,750	1.00 (reference)	1.00 (reference)	1.00 (reference)
Midday-afternoon	445	39,763	274,999	0.66 (0.57-0.77); 1.4e-7	0.69 (0.59-0.80); 2.2e-6	0.72 (0.62-0.85); 4.4e-5[a]
Evening	84	8009	55,706	0.93 (0.73-1.19); 0.57	0.94 (0.74-1.21); 0.64	0.85 (0.66-1.10); 0.21
Mixed	238	26,057	181,549	0.72 (0.60-0.86); 3.0e-4	0.74 (0.62-0.88); 7.6e-4	0.74 (0.62-0.89); 0.001[a]
**Cancer mortality**	1793	89,141	618,004			
Morning	345	15,312	105,750	1.00 (reference)	1.00 (reference)	1.00 (reference)
Midday-afternoon	852	39,763	274,999	0.96 (0.84-1.08); 0.47	0.97 (0.86-1.10); 0.63	0.97 (0.86-1.10); 0.67
Evening	147	8009	55,706	1.14 (0.94-1.39); 0.19	1.14 (0.94-1.39); 0.19	1.07 (0.88-1.31); 0.48
Mixed	449	26,057	181,549	0.96 (0.83-1.10); 0.54	0.96 (0.84-1.11); 0.61	0.96 (0.83-1.11); 0.60

[a] All *P* values remained significant after multiple testing with the FDR method. Cox proportional hazard regression was used to examine the associations. **Model 1** was adjusted for age and sex. **Model 2** was adjusted as in model 1 and for ethnicity, Townsend index of deprivation, recruitment center, education level, season of accelerometer wear, smoking status, and alcohol intake, healthy diet score. **Model 3** was adjusted as in model 2 and for sleep duration (< 7 hours, 7-8 hours, > 8 hours), sleep midpoint, and total MVPA volume. **CVD**: cardiovascular disease; **HR**: hazard ratio; **MVPA**: moderate to vigorous physical activity.

Supplementary Table 3. Sensitivity analysis on the associations between timing of MVPA and mortality risk by excluding participants with shift work history

Outcomes	Events	n	Person-years	Model 1<br>HR (95% CI); *P*	Model 2<br>HR (95% CI); *P*	Model 3<br>HR (95% CI); *P*[a]
**All-cause mortality**	2497	71,714	497,194			
Morning	531	12,361	85,331	1.00 (reference)	1.00 (reference)	1.00 (reference)
Midday-afternoon	1170	32,099	221,997	0.85 (0.76-0.94); 0.001	0.86 (0.78-0.96); 0.005	0.88 (0.80-0.98); 0.02[a]
Evening	198	6458	44,932	1.03 (0.87-1.21); 0.75	1.03 (0.87-1.21); 0.76	0.96 (0.81-1.13); 0.60
Mixed	598	20,796	144,934	0.85 (0.76-0.96); 6.9e-4	0.86 (0.76-0.97); 0.01	0.86 (0.77-0.97); 0.01[a]
**CVD mortality**	859	71,714	497,194			
Morning	221	12,361	85,331	1.00 (reference)	1.00 (reference)	1.00 (reference)
Midday-afternoon	373	32,099	221,997	0.65 (0.55-0.76); 2.7e-7	0.67 (0.57-0.79); 2.5e-6	0.70 (0.59-0.83); 4.1e-5[a]
Evening	67	6458	44,932	0.87 (0.66-1.14); 0.32	0.87 (0.66-1.15); 0.33	0.80 (0.60-1.05); 0.11
Mixed	198	20,796	144,934	0.70 (0.58-0.85); 3.4e-4	0.72 (0.59-0.97); 7.0e-4	0.73 (0.60-0.88); 0.001[a]
**Cancer mortality**	1511	71,714	497,194			
Morning	292	12,361	85,331	1.00 (reference)	1.00 (reference)	1.00 (reference)
Midday-afternoon	726	32,099	221,997	0.96 (0.83-1.10); 0.51	0.97 (0.85-1.11); 0.66	0.98 (0.85-1.12); 0.76
Evening	126	6458	44,932	1.16 (0.94-1.43); 0.17	1.16 (0.94-1.43); 0.17	1.10 (0.89-1.36); 0.40
Mixed	367	20,796	144,934	0.93 (0.80-1.09); 0.36	0.94 (0.80-1.10); 0.42	0.94 (0.80-1.09); 0.41

[a] All *P* values remained significant after multiple testing with the FDR method. Cox proportional hazard regression was used to examine the associations. **Model 1** was adjusted for age and sex. **Model 2** was adjusted as in model 1 and for ethnicity, Townsend index of deprivation, recruitment center, education level, season of accelerometer wear, smoking status, and alcohol intake, healthy diet score. **Model 3** was adjusted as in model 2 and for sleep duration (< 7 hours, 7-8 hours, > 8 hours), sleep midpoint, and total MVPA volume. **CVD**: cardiovascular disease; **HR**: hazard ratio; **MVPA**: moderate to vigorous physical activity.

Supplementary Table 4. Sensitivity analysis on the associations between timing of MVPA and mortality risk by excluding participants who wore accelerometers during the daylight saving time transition

Outcomes	Events	n	Person-years	Model 1 HR (95% CI); *P*	Model 2 HR (95% CI); *P*	Model 3 HR (95% CI); *P*[a]
**All-cause mortality**	1849	88,027	450,358			
Morning	394	15,132	77,198	1.00 (reference)	1.00 (reference)	1.00 (reference)
Midday-afternoon	844	39,213	200,043	0.87 (0.79-0.95); 0.004	0.89 (0.81-0.98); 0.01	0.90 (0.82-0.99); 0.04
Evening	154	7955	40,806	1.08 (0.93-1.25); 0.34	1.08 (0.93-1.26); 0.29	1.01 (0.86-1.17); 0.94
Mixed	457	25,727	132,311	0.88 (0.79-0.98); 0.02	0.89 (0.80-0.99); 0.04	0.89 (0.80-1.00); 0.04
**CVD mortality**	624	88,027	450358			
Morning	158	15,132	77,198	1.00 (reference)	1.00 (reference)	1.00 (reference)
Midday-afternoon	270	39,213	200,043	0.67 (0.57-0.78); 2.9e-7	0.69 (0.60-0.81); 3.4e-6	0.73 (0.63-0.85); 8.2e-5[a]
Evening	55	7955	40,806	0.97 (0.76-1.24); 0.82	0.99 (0.77-1.26); 0.93	0.90 (0.71-1.15); 0.42
Mixed	141	25,727	132,311	0.72 (0.60-0.86); 2.3e-4	0.73 (0.61-0.88); 6.6e-4	0.74 (0.62-0.89); 0.001[a]
**Cancer mortality**	1176	88,027	450358			
Morning	225	15,132	77,198	1.00 (reference)	1.00 (reference)	1.00 (reference)
Midday-afternoon	561	39,213	200,043	0.97 (0.85-1.10); 0.62	0.98 (0.87-1.12); 0.81	0.99 (0.87-1.12); 0.86
Evening	100	7955	40,806	1.16 (0.96-1.41); 0.13	1.17 (0.96-1.42); 0.12	1.10 (0.90-1.33); 0.35
Mixed	290	25,727	132,311	0.96 (0.83-1.11); 0.56	0.97 (0.84-1.12); 0.66	0.97 (0.84-1.12); 0.65

[a] All *P* values remained significant after multiple testing with the FDR method. Cox proportional hazard regression was used to examine the associations. **Model 1** was adjusted for age and sex. **Model 2** was adjusted as in model 1 and for ethnicity, Townsend index of deprivation, recruitment center, education level, season of accelerometer wear, smoking status, and alcohol intake, healthy diet score. **Model 3** was adjusted as in model 2 and for sleep duration (< 7 hours, 7-8 hours, > 8 hours), sleep midpoint, and total MVPA volume. **CVD**: cardiovascular disease; **HR**: hazard ratio; **MVPA**: moderate to vigorous physical activity.

Supplementary Table 5. Sensitivity analysis on the associations between timing of MVPA and mortality risk by controlling for month of accelerometer wear

Outcomes	Events	n	Person-years	Model 1<br>HR (95% CI); *P*	Model 2<br>HR (95% CI); *P*	Model 3<br>HR (95% CI); *P*[a]
**All-cause mortality**	3088	92,139	638,825			
Morning	652	15,865	109,537	1.00 (reference)	1.00 (reference)	1.00 (reference)
Midday-afternoon	1432	41,125	284,481	0.85 (0.78-0.94); 7.5e-4	0.87 (0.79-0.95); 0.003	0.89 (0.81-0.97); 0.01[a]
Evening	249	8307	57,768	1.05 (0.91-1.21); 0.54	1.05 (0.90-1.21); 0.53	0.97 (0.84-1.13); 0.71
Mixed	755	26,842	187,039	0.88 (0.79-0.97); 0.01	0.89 (0.80-0.98); 0.02	0.89 (0.80-0.99); 0.03[a]
**CVD mortality**	1076	92,139	638,825			
Morning	274	15,865	109,537	1.00 (reference)	1.00 (reference)	1.00 (reference)
Midday-afternoon	464	41,125	284,481	0.66 (0.57-0.77); 4.4e-8	0.68 (0.59-0.80); 7.0e-7	0.72 (0.62-0.84); 2.2e-5[a]
Evening	90	8307	57,768	0.94 (0.74-1.20); 0.64	0.96 (0.76-1.22); 0.75	0.87 (0.68-1.11); 0.27
Mixed	248	26,842	187,039	0.72 (0.60-0.85); 1.7e-4	0.74 (0.62-0.87); 4.9e-4	0.74 (0.62-0.88); 8.2e-4[a]
**Cancer mortality**	1872	92,139	638,825			
Morning	362	15,865	109,537	1.00 (reference)	1.00 (reference)	1.00 (reference)
Midday-afternoon	888	41,125	284,481	0.95 (0.84-1.08); 0.42	0.96 (0.85-1.09); 0.57	0.97 (0.86-1.10); 0.62
Evening	154	8307	57,768	1.14 (0.94-1.37); 0.19	1.13 (0.93-1.37); 0.21	1.06 (0.88-1.29); 0.53
Mixed	468	26,842	187,039	0.96 (0.83-1.10); 0.53	0.96 (0.84-1.10); 0.59	0.96 (0.84-1.10); 0.57

[a] All P values remained significant after multiple testing with the FDR method. Cox proportional hazard regression was used to examine the associations. Model 1 was adjusted for age and sex. Model 2 was adjusted as in model 1 and for ethnicity, Townsend index of deprivation, recruitment center, education level, **month of accelerometer wear**, healthy diet score, smoking status, and alcohol intake. Model 3 was adjusted as in model 2 and for sleep duration (< 7 hours, 7-8 hours, > 8 hours), sleep midpoint, and total MVPA volumes. **CVD**: cardiovascular disease; **HR**: hazard ratio; **MVPA**: moderate to vigorous physical activity.

Supplementary Table 6. Sensitivity analysis on the associations between timing of MVPA and mortality risk by additionally adjusting for health-related variables potentially on the causal pathway

Outcomes	Events	n	Person-years	Model 4<br>HR (95% CI); *P*[a]
**All-cause mortality**	3088	92,139	638,825	
Morning	652	15,865	109,537	1.00 (reference)
Midday-afternoon	1432	41,125	284,481	0.90 (0.82-0.99); 0.03
Evening	249	8307	57,768	0.99 (0.85-1.15); 0.90
Mixed	755	26,842	187,039	0.91 (0.82-1.02); 0.09
**CVD mortality**	1076	92,139	638,825	
Morning	274	15,865	109,537	1.00 (reference)
Midday-afternoon	464	41,125	284,481	0.73 (0.63-0.85); 5.8e-5[a]
Evening	90	8307	57,768	0.89 (0.70-1.13); 0.32
Mixed	248	26,842	187,039	0.77 (0.65-0.92); 0.004[a]
**Cancer mortality**	1872	92,139	638,825	
Morning	362	15,865	109,537	1.00 (reference)
Midday-afternoon	888	41,125	284,481	0.98 (0.87-1.11); 0.80
Evening	154	8,307	57,768	1.09 (0.90-1.32); 0.38
Mixed	468	26,842	187,039	0.98 (0.86-1.13); 0.83

[a] All *P* values remained significant after multiple testing with the FDR method. Cox proportional hazard regression was used to examine the associations. **Model 4** was adjusted as in model 3 and for Obesity, diabetes history, longstanding illness, depression history, CVD, and cancer history. **CVD**: cardiovascular disease; **HR**: hazard ratio; **MVPA**: moderate to vigorous physical activity.

Supplementary Table 7. Sensitivity analysis on the associations between timing of MVPA and mortality risk by excluding events within the first year of follow-up

Outcomes	Events	n	Person-years	Model 1 HR (95% CI); *P*	Model 2 HR (95% CI); *P*	Model 3 HR (95% CI); *P*[a]
**All-cause mortality**	2887	91,938	638,710			
Morning	604	15,817	109,509	1.00 (reference)	1.00 (reference)	1.00 (reference)
Midday-afternoon	1347	41,040	284,432	0.87 (0.79-0.95); 0.003	0..88 (0.80-0.97); 0.01	0.89 (0.81-0.99); 0.03
Evening	227	8285	57,756	1.03 (0.88-1.20); 0.70	1.04 (0.89-1.21); 0.65	0.96 (0.82-1.12); 0.58
Mixed	709	26,796	187,013	0.89 (0.80-0.99); 0.03	0.90 (0.81-1.0); 0.05	0.90 (0.80-1.00); 0.05
**CVD mortality**	997	92,060	638,710			
Morning	253	15,844	109,525	1.00 (reference)	1.00 (reference)	1.00 (reference)
Midday-afternoon	430	41,091	284,464	0.66 (0.57-0.77); 1.8e-7	0.69 (0.59-0.80); 2.3e-6	0.72 (0.61-0.84); 3.8e-5[a]
Evening	80	8297	57,763	0.91 (0.71-1.17); 0.48	0.93 (0.72-1.19); 0.56	0.84 (0.64-1.08); 0.17
Mixed	234	26,828	187,032	0.73 (0.61-0.88); 7.2e-4	0.75 (0.63-0.90); 0.002	0.75 (0.63-0.90); 0.002[a]
**Cancer mortality**	1752	92,019	638,753			
Morning	335	15,838	109,520	1.00 (reference)	1.00 (reference)	1.00 (reference)
Midday-afternoon	837	41,074	284,449	0.97 (0.85-1.10); 0.62	0.98 (0.87-1.12); 0.78	0.98 (0.86-1.11); 0.75
Evening	140	8293	57,760	1.11 (0.91-1.36); 0.29	1.11 (0.91-1.36); 0.29	1.05 (0.86-1.28); 0.64
Mixed	440	26,814	187,024	0.97 (0.84-1.12); 0.67	0.98 (0.85-1.13); 0.75	0.97 (0.84-1.12); 0.69

[a] All P values remained significant after multiple testing with the FDR method. Cox proportional hazard regression was used to examine the associations. Model 1 was adjusted for age and sex. Model 2 was adjusted as in model 1 and for ethnicity, Townsend index of deprivation, recruitment center, education level, season of accelerometer wear, smoking status, and alcohol intake, healthy diet score. Model 3 was adjusted as in model 2 and for sleep duration (< 7 hours, 7-8 hours, > 8 hours), sleep midpoint, and total MVPA volume. CVD: cardiovascular disease; HR: hazard ratio; MVPA: moderate to vigorous physical activity.

Supplementary Table 8. Sensitivity analysis on the associations between timing of MVPA and mortality risk by censoring up to Dec 31, 2019

Outcomes	Events	n	Person-years	Model 1<br>HR (95% CI); *P*	Model 2<br>HR (95% CI); *P*	Model 3<br>HR (95% CI); *P*[a]
**All-cause mortality**	1942	92,139	471,521			
Morning	418	15,865	80,917	1.00 (reference)	1.00 (reference)	1.00 (reference)
Midday-afternoon	882	41,125	209,883	0..82 (0.73-0.92); 7.8e-4	0.84 (0.75-0.94); 0.002	0.86 (0.76-0.96); 0.01[a]
Evening	158	8307	42,640	1.02 (0.85-1.23); 0.84	1.02 (0.85-1.23); 0.85	0.95 (0.79-1.14); 0.58
Mixed	484	26,842	138,081	0.87 (0.76-0.99); 0.04	0.88 (0.77-1.00); 0.05	0.88 (0.77-1.01); 0.06
**CVD mortality**	654	92,139	471,521			
Morning	166	15,865	80,917	1.00 (reference)	1.00 (reference)	1.00 (reference)
Midday-afternoon	281	41,125	209,883	0.66 (0.54-0.80); 1.9e-5	0.68 (0.56-0.83); 9.4e-5	0.71 (0.59-0.87); 7.0e-4[a]
Evening	56	8307	42,640	0.95 (0.70-1.29); 0.73	0.96 (0.70-1.30); 0.77	0.87 (0.64-1.18); 0.36
Mixed	151	26,842	138,081	0.71 (0.57-0.89); 0.003	0.73 (0.58-0.91); 0.005	0.74 (0.59-0.92); 0.007[a]
**Cancer mortality**	1231	92,139	471,521			
Morning	239	15,865	80,917	1.00 (reference)	1.00 (reference)	1.00 (reference)
Midday-afternoon	583	41,125	209,883	0.95 (0.81-1.10); 0.48	0.97 (0.83-1.12); 0.65	0.98 (0.84-1.14); 0.78
Evening	103	8307	42,640	1.14 (0.91-1.44); 0.26	1.14 (0.91-1.44); 0.26	1.08 (0.85-1.36); 0.53
Mixed	306	26,842	138,081	0.95 (0.80-1.12); 0.52	0.95 (0.80-1.13); 0.60	0.95 (0.80-1.13); 0.60

[a] All *P* values remained significant after multiple testing with the FDR method. Cox proportional hazard regression was used to examine the associations. **Model 1** was adjusted for age and sex. **Model 2** was adjusted as in model 1 and for ethnicity, Townsend index of deprivation, recruitment center, education level, season of accelerometer wear, smoking status, and alcohol intake, healthy diet score. **Model 3** was adjusted as in model 2 and for sleep duration (< 7 hours, 7-8 hours, > 8 hours), sleep midpoint, and total MVPA volume. **CVD**: cardiovascular disease; **HR**: hazard ratio; **MVPA**: moderate to vigorous physical activity. 31 Dec 2019 is considered as the start of the COVID-19 pandemic [Xue et al. J Pineal Res. 2022].

Supplementary Table 9. Sensitivity analysis on the associations between timing of MVPA and mortality risk by using the subsample with ⩾6 days of accelerometer wear

Outcomes	Events	n	Person-years	Model 1 HR (95% CI); *P*	Model 2 HR (95% CI); *P*	Model 3 HR (95% CI); *P*[a]
**All-cause mortality**	1789	83,785	428,714			
Morning	376	14,142	72,180	1.00 (reference)	1.00 (reference)	1.00 (reference)
Midday-afternoon	823	37,712	192,391	0.87 (0.79-0.95); 0.004	0.88 (0.80-0.97); 0.01	0.90 (0.82-0.99); 0.04
Evening	135	7197	36,937	1.03 (0.88-1.21); 0.70	1.03 (0.88-1.21); 0.68	0.95 (0.81-1.12); 0.54
Mixed	455	24,734	127,206	0.89 (0.79-0.99); 0.03	0.90 (0.80-1.00); 0.05	0.90 (0.80-1.00); 0.06
**CVD mortality**	608	83,785	428,714			
Morning	150	14,142	72,180	1.00 (reference)	1.00 (reference)	1.00 (reference)
Midday-afternoon	265	37,712	192,391	0.67 (0.57-0.78); 4.5e-7	0.70 (0.60-0.81); 5.1e-6	0.73 (0.62-0.86); 9.7e-5[a]
Evening	48	7197	36,937	0.91 (0.70-1.17); 0.45	0.92 (0.71-1.19); 0.51	0.83 (0.64-1.07); 0.15
Mixed	145	24,734	127,206	0.72 (0.60-0.87); 4.0e-4	0.74 (0.62-0.89); 0.001	0.75 (0.62-0.90); 0.002[a]
**Cancer mortality**	1131	83,785	428,714			
Morning	216	14,142	72,180	1.00 (reference)	1.00 (reference)	1.00 (reference)
Midday-afternoon	543	37,712	192,391	0.96 (0.84-1.09); 0.53	0.97 (0.86-1.11); 0.69	0.98 (0.86-1.11); 0.74
Evening	85	7197	36,937	1.08 (0.88-1.32); 0.48	1.08 (0.88-1.32); 0.48	1.01 (0.82-1.24); 0.94
Mixed	287	24,734	127,206	0.97 (0.84-1.12); 0.65	0.98 (0.84-1.13); 0.73	0.97 (0.84-1.13); 0.71

[a] All *P* values remained significant after multiple testing with the FDR method. Cox proportional hazard regression was used to examine the associations. **Model 0** was the crude model. **Model 1** was adjusted for age and sex. **Model 2** was adjusted as in model 1 and for ethnicity, Townsend index of deprivation, recruitment center, education level, season of accelerometer wear, smoking status, and alcohol intake, healthy diet score. **Model 3** was adjusted as in model 2 and for sleep duration (< 7 hours, 7-8 hours, > 8 hours), sleep midpoint, and total MVPA volume. **CVD**: cardiovascular disease; **HR**: hazard ratio; **MVPA**: moderate to vigorous physical activity.

Supplementary Table 10. Interaction effects of timing of MVPA and age categories (< 65 and ≥ 65 years) on mortality risk

Outcomes	Multiplicative interaction HR (95% CI); *P*	Additive interaction		
		RERI (95% CI)	AP (95% CI)	S (95% CI)
**All-cause mortality**				
Midday-afternoon/Mixed (vs. Morning/Evening) & Older age (⩾ 65 vs. < 65 years)	1.16 (0.97-1.39); 0.10	**0.47 (0.15-0.80)**	**0.14 (0.05-0.23)**	**1.25 (1.07-1.47)**
**CVD mortality**				
Midday-afternoon/Mixed (vs. Morning/Evening) & Older age (⩾ 65 vs. < 65 years)	**1.38 (1.01-1.89); 0.04**	**1.34 (0.70-1.98)**	**0.30 (0.18-0.41)**	**1.61 (1.27-2.05)**
**Cancer mortality**				
Midday-afternoon/Mixed (vs. Morning/Evening) & Older age (⩾ 65 vs. < 65 years)	1.04 (0.83-1.30); 0.71	0.16 (-0.23-0.56)	0.06 (-0.07-0.19)	1.09 (0.88-1.36)

Cox proportional hazard regression was used to examine the associations of MVPA timing and age with mortality risk, which were adjusted for sex, ethnicity, Townsend index of deprivation, recruitment center, education level, season of accelerometer wear, smoking status, alcohol intake, healthy diet score, sleep duration (< 7 hours, 7-8 hours, > 8 hours), sleep midpoint, and total MVPA volume. Multiplicative and additive interaction analyses were performed to examine the interaction effects. **AP:** attributable proportion due to interaction; **CI:** confidence interval; **CVD**: cardiovascular disease; **HR**: hazard ratio; **MVPA**: moderate to vigorous physical activity; **RERI:** relative excess risk due to interaction; **S**: synergy index. According to their associations with mortality (table 2), four timing groups were combined into two groups: 1) midday-afternoon/mixed; 2) morning/evening. This combination method facilitated the analysis and interpretation of multiplicative and additive interaction effects, which was widely used in previous studies [Shan et al. BMJ, 2018.; Huang et al. Br J Sports Med. 2021].

Supplementary Table 11. Interaction effects of timing of MVPA and sex on mortality risk

Outcomes	Multiplicative interaction HR (95% CI); *P*	Additive interaction		
		RERI (95% CI)	AP (95% CI)	S (95% CI)
**All-cause mortality**				
Midday-afternoon/Mixed (vs. Morning/Evening) & Male	1.03 (0.88-1.20); 0.76	0.12 (-0.11-0.35)	0.06 (-0.05-0.18)	1.15 (0.88-1.50)
**CVD mortality**				
Midday-afternoon/Mixed (vs. Morning/Evening) & Male	1.07 (0.82-1.41); 0.62	**0.63 (0.095-1.17)**	**1.35 (1.04-1.77)**	**1.35 (1.04-1.77)**
**Cancer mortality**				
Midday-afternoon/Mixed (vs. Morning/Evening) & Male	0.93 (0.76-1.14); 0.51	-0.06 (-0.33-0.20)	-0.04 (-0.22-0.14)	0.89 (0.56-1.43)

Cox proportional hazard regression was used to examine the associations of MVPA timing and sex with mortality risk, which were adjusted for age, ethnicity, Townsend index of deprivation, recruitment center, education level, season of accelerometer wear, smoking status, alcohol intake, healthy diet score, sleep duration (< 7 hours, 7-8 hours, > 8 hours), sleep midpoint, and total MVPA volume. Multiplicative and additive interaction analyses were performed to examine the interaction effects. **AP:** attributable proportion due to interaction; **CI:** confidence interval; **CVD**: cardiovascular disease; **HR**: hazard ratio; **MVPA**: moderate to vigorous physical activity; **RERI:** relative excess risk due to interaction; **S**: synergy index. According to their associations with mortality (table 2), four timing groups were combined into two groups: 1) midday-afternoon/mixed; 2) morning/evening. This combination method facilitated the analysis and interpretation of multiplicative and additive interaction effects, which was widely used in previous studies [Shan et al. BMJ, 2018.; Huang et al. Br J Sports Med. 2021].

Supplementary Table 12. Interaction effects of timing of MVPA and MVPA level on mortality risk

Outcomes	Multiplicative interaction HR (95% CI); *P*	Additive interaction		
		RERI (95% CI)	AP (95% CI)	S (95% CI)
**All-cause mortality**				
Midday-afternoon/Mixed (vs. Morning/Evening) & Below WHO recommendation[a]	**1.30 (1.07-1.58); 0.009**	**0.40 (0.12-0.68)**	**0.25 (0.10-0.40)**	**1.66 (1.34-2.05)**
**CVD mortality**				
Midday-afternoon/Mixed (vs. Morning/Evening) & Below WHO recommendation[a]	1.31 (0.94-1.83); 0.11	**0.65 (0.22-1.07)**	**0.26 (0.10-0.43)**	**1.80 (1.07-3.03)**
**Cancer mortality**				
Midday-afternoon/Mixed (vs. Morning/Evening) & Below WHO recommendation[a]	1.20 (0.94-1.53); 0.14	0.24 (-0.08-0.56)	0.16 (-0.03-0.36)	1.49 (1.05-2.11)

[a] WHO recommendation: At least 150 minutes of moderate-intensity aerobic physical activity throughout the week, or at least 75 minutes of vigorous-intensity aerobic physical activity throughout the week, or an equivalent combination of moderate- and vigorous-intensity activity.

Cox proportional hazard regression was used to examine the associations of MVPA timing and MVPA level with mortality risk, which were adjusted for age, sex, ethnicity, Townsend index of deprivation, recruitment center, education level, season of accelerometer wear, smoking status, alcohol intake, healthy diet score, sleep duration (< 7 hours, 7-8 hours, > 8 hours), and sleep midpoint. Multiplicative and additive interaction analyses were performed to examine the interaction effects. **AP:** attributable proportion due to interaction; **CI:** confidence interval; **CVD**: cardiovascular disease; **HR**: hazard ratio; **MVPA**: moderate to vigorous physical activity; **RERI:** relative excess risk due to interaction; **S**: synergy index. According to their associations with mortality (table 2), four timing groups were combined into two groups: 1) midday-afternoon/mixed; 2) morning/evening. This combination method facilitated the analysis and interpretation of multiplicative and additive interaction effects, which was widely used in previous studies [Shan et al. BMJ, 2018.; Huang et al. Br J Sports Med. 2021].

Supplementary Table 13. Interaction effects of timing of MVPA and CVDs on mortality risk

Outcomes	Multiplicative interaction HR (95% CI); *P*	Additive interaction		
		RERI (95% CI)	AP (95% CI)	S (95% CI)
**All-cause mortality**				
Midday-afternoon/Mixed (vs. Morning/Evening) & CVDs	**1.17 (1.00-1.37); 4.7e-2**	**0.29 (0.07-0.52)**	**0.16 (0.04-0.27)**	**1.51 (1.07-2.12)**
**CVD mortality**				
Midday-afternoon/Mixed (vs. Morning/Evening) & CVDs	**1.32 (1.01-1.71); 0.04**	**0.94 (0.42-1.47)**	**0.28 (0.15-0.42)**	**1.68 (1.24-2.28)**
**Cancer mortality**				
Midday-afternoon/Mixed (vs. Morning/Evening) & CVDs	1.09 (0.88-1.34); 0.43	0.12 (-0.14-0.38)	0.08 (-0.09-0.25)	1.36 (0.69-2.69)

Cox proportional hazard regression was used to examine the associations of MVPA timing and CVDs with mortality risk, which were adjusted for age, sex, ethnicity, Townsend index of deprivation, recruitment center, education level, season of accelerometer wear, smoking status, alcohol intake, healthy diet score, sleep duration (< 7 hours, 7-8 hours, > 8 hours), sleep midpoint, and total MVPA volume. Multiplicative and additive interaction analyses were performed to examine the interaction effects. **AP:** attributable proportion due to interaction; **CI:** confidence interval; **CVDs**: cardiovascular diseases; **HR**: hazard ratio; **MVPA**: moderate to vigorous physical activity; **RERI:** relative excess risk due to interaction; **S**: synergy index. According to their associations with mortality (table 2), four timing groups were combined into two groups: 1) midday-afternoon/mixed; 2) morning/evening. This combination method facilitated the analysis and interpretation of multiplicative and additive interaction effects, which was widely used in previous studies [Shan et al. BMJ, 2018.; Huang et al. Br J Sports Med. 2021].

Supplementary Table 14. Interaction effects of timing of MVPA and obesity on mortality risk

Outcomes	Multiplicative interaction HR (95% CI); *P*	Additive interaction		
		RERI (95% CI)	AP (95% CI)	S (95% CI)
**All-cause mortality**				
Midday-afternoon/Mixed (vs. Morning/Evening) & Obesity	1.07 (0.90-1.27); 0.43	0.13 (-0.10-0.35)	0.08 (-0.06-0.23)	1.33 (0.80-2.22)
**CVD mortality**				
Midday-afternoon/Mixed (vs. Morning/Evening) & Obesity	1.11 (0.85-1.44); 0.45	0.34 (-0.09-0.78)	0.16 (-0.03-0.35)	1.44 (0.89-2.33)
**Cancer mortality**				
Midday-afternoon/Mixed (vs. Morning/Evening) & Obesity	1.09 (0.87-1.36); 0.45	0.12 (-0.16-0.40)	0.09(-0.10-0.28)	1.45 (0.60-3.50)

Cox proportional hazard regression was used to examine the associations of MVPA timing and obesity with mortality risk, which were adjusted for age, sex, ethnicity, Townsend index of deprivation, recruitment center, education level, season of accelerometer wear, smoking status, alcohol intake, healthy diet score, sleep duration (< 7 hours, 7-8 hours, > 8 hours), sleep midpoint, and total MVPA volume. Multiplicative and additive interaction analyses were performed to examine the interaction effects. **AP:** attributable proportion due to interaction; **CI:** confidence interval; **CVD**: cardiovascular disease; **HR**: hazard ratio; **MVPA**: moderate to vigorous physical activity; **RERI:** relative excess risk due to interaction; **S**: synergy index. According to their associations with mortality (table 2), four timing groups were combined into two groups: 1) midday-afternoon/mixed; 2) morning/evening. This combination method facilitated the analysis and interpretation of multiplicative and additive interaction effects, which was widely used in previous studies [Shan et al. BMJ, 2018.; Huang et al. Br J Sports Med. 2021].

Supplementary Table 15. Subgroup analysis on the associations between timing of MVPA and mortality risk stratified by age categories (< 65 and ≥ 65 years)

Outcomes	Age categories	Events/n	Person-years	Model 1 HR (95% CI); *P*	Model 2 HR (95% CI); *P*	Model 3 HR (95% CI); *P*[a]
**All-cause mortality**						
Midday-afternoon/Mixed (vs. Morning/Evening)	< 65 years	826/51,914	364,244	0.93 (0.79-1.08); 0.33	0.93 (0.80-1.09); 0.36	0.94 (0.80-1.09); 0.40
	⩾ 65 years	2262/40,225	274,582	0.83 (0.75-0.90); 3.2e-5	0.84 (0.77-0.92); 1.9e-4	0.88 (0.81-0.97); 0.007[a]
**CVD mortality**						
Midday-afternoon/Mixed (vs. Morning/Evening)	< 65 years	244/51,914	364,244	0.87 (0.66-1.15); 0.32	0.88 (0.67-1.16); 0.37	0.92 (0.70-1.22); 0.56
	⩾ 65 years	832/40,225	274,582	0.65 (0.56-0.75); 2.5e-9	0.67 (0.58-0.77); 3.5e-8	0.71 (0.62-0.82); 4.0e-6[a]
**Cancer mortality**						
Midday-afternoon/Mixed (vs. Morning/Evening)	< 65 years	543/51,914	364,244	0.92 (0.76-1.11); 0.38	0.92 (0.76-1.12); 0.41	0.93 (0.77-1.12); 0.42
	⩾ 65 years	1329/40,225	274,582	0.91 (0.81-1.03); 0.15	0.93 (0.82-1.05); 0.23	0.96 (0.85-1.08); 0.48

[a] All *P* values remained significant after multiple testing with the FDR method. Cox proportional hazard regression was used to examine the associations. **Model 1** was adjusted for age (continuous) and sex. **Model 2** was adjusted as in model 1 and for ethnicity, Townsend index of deprivation, recruitment center, education level, season of accelerometer wear, smoking status, alcohol intake, and healthy diet score. **Model 3** was adjusted as in model 2 and for sleep duration (< 7 hours, 7-8 hours, > 8 hours), sleep midpoint, and total MVPA volume. **CVD**: cardiovascular disease; **HR**: hazard ratio; **MVPA**: moderate to vigorous physical activity. According to their associations with mortality (table 2), four timing groups were combined into two groups: 1) midday-afternoon/mixed; 2) morning/evening. This combination method facilitated the analysis and interpretation of multiplicative and additive interaction effects, which was widely used in previous studies [Shan et al. BMJ, 2018.; Huang et al. Br J Sports Med. 2021].

Supplementary Table 16. Subgroup analysis on the associations between timing of MVPA and mortality risk stratified by sex

Outcomes	Sex categories	Events/n	Person-years	Model 1 HR (95% CI); *P*	Model 2 HR (95% CI); *P*	Model 3 HR (95% CI); *P*[a]
**All-cause mortality**						
Midday-afternoon/Mixed (vs. Morning/Evening)	Female	1264/52,045	362,306	0.87 (0.77-0.98); 0.24	0.87 (0.77-0.99); 0.03	0.90 (0.80-1.02); 0.10
	Male	1824/40,094	276,519	0.84 (0.76-0.93); 5.4e-4	0.85 (0.77-0.95); 0.002	0.89 (0.80-0.98); 0.02
**CVD mortality**						
Midday-afternoon/Mixed (vs. Morning/Evening)	Female	346/52,045	362,306	0.73 (0.58-0.91); 0.006	0.74 (0.59-0.92); 0.007	0.79 (0.63-0.99); 0.04
	Male	730/40,094	276,519	0.67 (0.57-0.78); 2.5e-7	0.69 (0.59-0.81); 2.3e-6	0.74 (0.63-0.86); 10e-5[a]
**Cancer mortality**						
Midday-afternoon/Mixed (vs. Morning/Evening)	Female	841/52,045	362,306	0.89 (0.77-1.03); 0.13	0.89 (0.77-1.04); 0.14	0.91 (0.78-1.06); 0.22
	Male	1031/40,094	276,519	0.94 (0.82-1.08); 0.41	0.96 (0.84-1.10); 0.57	0.98 (0.85-1.13); 0.78

[a] All *P* values remained significant after multiple testing with the FDR method. Cox proportional hazard regression was used to examine the associations. **Model 1** was adjusted for age. **Model 2** was adjusted as in model 1 and for ethnicity, Townsend index of deprivation, recruitment center, education level, season of accelerometer wear, smoking status, alcohol intake, and healthy diet score. **Model 3** was adjusted as in model 2 and for sleep duration (< 7 hours, 7-8 hours, > 8 hours), sleep midpoint, and total MVPA volume. **CVD**: cardiovascular disease; **HR**: hazard ratio; **MVPA**: moderate to vigorous physical activity. According to their associations with mortality (table 2), four timing groups were combined into two groups: 1) midday-afternoon/mixed; 2) morning/evening. This combination method facilitated the analysis and interpretation of multiplicative and additive interaction effects, which was widely used in previous studies [Shan et al. BMJ, 2018.; Huang et al. Br J Sports Med. 2021].

Supplementary Table 17. Subgroup analysis on the associations between timing of MVPA and mortality risk stratified by MVPA levels

Outcomes	PA levels	Events/n	Person-years	Model 1 HR (95% CI); *P*	Model 2 HR (95% CI); *P*	Model 3 HR (95% CI); *P*[b]
**All-cause mortality**						
Midday-afternoon/Mixed (vs. Morning/Evening)	Meets WHO recommendation[a]	727/36,013	251,700	1.06 (0.89-1.26); 0.51	1.06 (0.89-1.26); 0.50	1.06 (0.89-1.27); 0.49
	Below WHO recommendation[a]	2361/56,126	387,127	0.82 (0.75-0.89); 6.5e-6	0.83 (0.76-0.91); 3.1e-5	0.89 (0.81-0.97); 0.008[b]
**CVD mortality**						
Midday-afternoon/Mixed (vs. Morning/Evening)	Meets WHO recommendation[a]	218/36,013	251,700	0.88 (0.65-1.19); 0.40	0.88 (0.65-1.20); 0.42	0.89 (0.66-1.21); 0.47
	Below WHO recommendation[a]	858/56,126	387,127	0.67 (0.58-0.77); 1.6e-8	0.69 (0.60-0.79); 1.7e-7	0.77 (0.66-0.88); 2.0e-4[b]
**Cancer mortality**						
Midday-afternoon/Mixed (vs. Morning/Evening)	Meets WHO recommendation[a]	478/36,013	251,700	1.07 (0.86-1.32); 0.55	1.07 (0.86-1.32); 0.56	1.06 (0.85-1.32); 0.59
	Below WHO recommendation[a]	1394/56,126	387,127	0.89 (0.80-1.00); 0.06	0.90 (0.80-1.01); 0.08	0.94 (0.84-1.05); 0.29

[a] WHO recommendation: At least 150 minutes of moderate-intensity aerobic physical activity throughout the week, or at least 75 minutes of vigorous-intensity aerobic physical activity throughout the week, or an equivalent combination of moderate- and vigorous-intensity activity. **[b]** All *P* values remained significant after multiple testing with the FDR method.

Cox proportional hazard regression was used to examine the associations. **Model 1** was adjusted for age and sex. **Model 2** was adjusted as in model 1 and for ethnicity, Townsend index of deprivation, recruitment center, education level, season of accelerometer wear, smoking status, alcohol intake, and healthy diet score. **Model 3** was adjusted as in model 2 and for sleep duration (< 7 hours, 7-8 hours, > 8 hours), sleep midpoint, and total MVPA volume. **CVD**: cardiovascular disease; **HR**: hazard ratio; **MVPA**: moderate to vigorous physical activity. According to their associations with mortality (table 2), four timing groups were combined into two groups: 1) midday-afternoon/mixed; 2) morning/evening. This combination method facilitated the analysis and interpretation of multiplicative and additive interaction effects, which was widely used in previous studies [Shan et al. BMJ, 2018.; Huang et al. Br J Sports Med. 2021]

Supplementary Table 18. Subgroup analysis on the associations between timing of MVPA and mortality risk stratified by CVDs

Outcomes	CVD categories	Events/n	Person-years	Model 1 HR (95% CI); *P*	Model 2 HR (95% CI); *P*	Model 3 HR (95% CI); *P*[a]
**All-cause mortality**						
Midday-afternoon/Mixed (vs. Morning/Evening)	No CVDs[a]	1705/69,478	484,565	0.93 (0.84-1.04); 0.20	0.94 (0.84-1.04); 0.24	0.96 (0.86-1.07); 0.42
	CVDs[a]	1383/22,661	154,261	0.78 (0.70-0.87); 1.6e-5	0.80 (0.71-0.89); 9.5e-5	0.84 (0.75-0.94); 0.002[a]
**CVD mortality**						
Midday-afternoon/Mixed (vs. Morning/Evening)	No CVDs[a]	449/69,478	484,565	0.84 (0.68-1.03); 0.09	0.85 (0.69-1.04); 0.11	0.89 (0.72-1.09); 0.26
	CVDs[a]	627/22,661	154,261	0.62 (0.53-0.73); 7.9e-9	0.64 (0.55-0.76); 1.1e-7	0.69 (0.58-0.81); 6.1e-6[a]
**Cancer mortality**						
Midday-afternoon/Mixed (vs. Morning/Evening)	No CVDs[a]	1137/69,478	484,565	0.96 (0.84-1.10); 0.56	0.97 (0.85-1.10); 0.62	0.98 (0.85-1.12); 0.72
	CVDs[a]	735/22,661	154,261	0.87 (0.75-1.02); 0.09	0.89 (0.76-1.04); 0.15	0.92 (0.78-1.07); 0.27

[a] All *P* values remained significant after multiple testing with the FDR method.

Cox proportional hazard regression was used to examine the associations. **Model 1** was adjusted for age and sex. **Model 2** was adjusted as in model 1 and for ethnicity, Townsend index of deprivation, recruitment center, education level, season of accelerometer wear, smoking status, alcohol intake, and healthy diet score. **Model 3** was adjusted as in model 2 and for sleep duration (< 7 hours, 7-8 hours, > 8 hours), sleep midpoint, and total MVPA volume. **CVDs**: cardiovascular diseases; **HR**: hazard ratio; **MVPA**: moderate to vigorous physical activity. According to their associations with mortality (table 2), four timing groups were combined into two groups: 1) midday-afternoon/mixed; 2) morning/evening. This combination method facilitated the analysis and interpretation of multiplicative and additive interaction effects, which was widely used in previous studies [Shan et al. BMJ, 2018.; Huang et al. Br J Sports Med. 2021]

Supplementary Table 19. Subgroup analysis on the associations between timing of MVPA and mortality risk stratified by obesity

Outcomes	Obesity categories	Events/n	Person-years	Model 1<br>HR (95% CI); *P*	Model 2<br>HR (95% CI); *P*	Model 3<br>HR (95% CI); *P*[a]
**All-cause mortality**						
Midday-afternoon/Mixed (vs. Morning/Evening)	No obesity[a]	901/24,172	167306	0.88 (0.80-0.97); 0.008	0.89 (0.81-0.98); 0.02	0.92 (0.84-1.01); 0.08
	Obesity[a]	2187/67,967	471521	0.84 (0.73-0.97); 0.01	0.85 (0.74-0.98); 0.02	0.87 (0.75-1.00); 4.7e-2
**CVD mortality**						
Midday-afternoon/Mixed (vs. Morning/Evening)	No obesity[a]	364/24,172	167306	0.73 (0.63-0.86); 1.3e-4	0.75 (0.64-0.88); 4.6e-4	0.76 (0.68-0.93); 0.005[a]
	Obesity[a]	712/67,967	471521	0.68 (0.55-0.83); 2.3e-4	0.68 (0.56-0.84); 4.0e-4	0.72 (0.59-0.90); 0.003[a]
**Cancer mortality**						
Midday-afternoon/Mixed (vs. Morning/Evening)	No obesity[a]	516/24,172	167306	0.95 (0.85-1.08); 0.44	0.96 (0.85-1.09); 0.54	0.97 (0.86-1.10); 0.67
	Obesity[a]	1356/67,967	471521	0.89 (0.74-1.08); 0.23	0.90 (0.74-1.08); 0.25	0.92 (0.76-1.11); 0.38

[a] All *P* values remained significant after multiple testing with the FDR method.

Cox proportional hazard regression was used to examine the associations. **Model 1** was adjusted for age and sex. **Model 2** was adjusted as in model 1 and for ethnicity, Townsend index of deprivation, recruitment center, education level, season of accelerometer wear, smoking status, alcohol intake, and healthy diet score. **Model 3** was adjusted as in model 2 and for sleep duration (< 7 hours, 7-8 hours, > 8 hours), sleep midpoint, and total MVPA volume. **CVD**: cardiovascular disease; **HR**: hazard ratio; **MVPA**: moderate to vigorous physical activity. According to their associations with mortality (table 2), four timing groups were combined into two groups: 1) midday-afternoon/mixed; 2) morning/evening. This combination method facilitated the analysis and interpretation of multiplicative and additive interaction effects, which was widely used in previous studies [Shan et al. BMJ, 2018.; Huang et al. Br J Sports Med. 2021]

Supplementary Table 20. The information sources of exposure, outcomes, and covariates

Variables	Touchscreen questionnaire		Hospital records	Cancer Registry	Death Registry	Accelerometer
	Initial assessment	Repeated assessments				
All-cause mortality	-	-	-	-	A00-U89	-
Cardiovascular disease mortality	-	-	-	-	I00–I99	-
Cancer mortality	-	-	-	-	C00-C97	-
Age at accelerometry	Field ID 34&52	-	-	-	-	Field ID 90010
Sex	Field ID 31	-	-	-	-	-
Ethnicity	Field ID 21000	-	-	-	-	-
Townsend deprivation index	Field ID 189	-	-	-	-	-
Recruitment regions	Field ID 54	-	-	-	-	-
Education level	Field ID 6138	Field ID 6138				
Season of accelerometer wear	-	-	-	-	-	Field ID 90010
Smoking status	Field ID 20116	Field ID 20116	-	-	-	-
Alcohol consumption	Field ID 1558	Field ID 1558	-	-	-	-
Healthy diet score	Field ID 1289/1299/1309/1319/1329/1339/1349/1369/1379/1389	Field ID 1289/1299/1309/1319/1329/1339/1349/1369/1379/1389	-	-	-	-
Sleep duration	-	-	-	-	-	Return ID 1862
Sleep midpoint (hh:mm)	-	-	-	-	-	Return ID 1862
Health status						
Obesity	Field ID 21001	Field ID 21001	-	-	-	-
Diabetes history	Field ID 2443	Field ID 2443	ICD E10-E14		ICD E10-E14	
Longstanding illness	Field ID 2188	Field ID 2188	-	-	-	-
Depression history	Field ID 20123/20124/20125	-	ICD F32/33	-	ICD F32/33	-
Cardiovascular disease history	-	-	I00–I99	-	I00–I99	-
Cancer history	Field ID 2453	Field ID 2453	ICD C00-C97	ICD C00-C97	ICD C00-C97	

Supplementary Table 21. The numbers (percentages) of participants with missing covariates

Covariates	n	%
Any missing covariate	2998	3.25
Age at accelerometry	0	0
Sex	0	0
White ethnicity	322	0.35
Townsend deprivation index	103	0.11
Recruitment regions	0	0
Education level	529	0.57
Season of accelerometer wear	0	0
Smoking status	208	0.23
Alcohol consumption	63	0.07
Healthy diet score	41	0.04
Sleep duration	212	0.23
Sleep midpoint	212	0.23
Health status		
Obesity	170	0.18
Diabetes history	0	0
Longstanding illness	1478	1.60
Depression history	0	0
Cardiovascular disease history	0	0
Cancer history	161	0.17

Supplementary Table 22. Baseline characteristics of overall sample and complete case sample

Characteristics	Overall sample [dataset with imputation] (n = 92,139)	Complete case sample [excluding those with any missing covariates from overall sample] (n = 89,141)
**Age at accelerometry (years)**	62.38 ± 7.84	62.35 ± 7.83
**Sex (female/male)**	52,045/40,094	50,233/38,908
**White ethnicity**	89,323 (96.94)	86,502 (97.04)
**Townsend deprivation index, median [IQR]**	-2.45 [3.63]	-2.46 [3.61]
**Recruitment regions**		
England	82,716 (89.77)	80,013 (89.76)
Wales	3449 (3.74)	3343 (3.75)
Scotland	5974 (6.48)	5785 (6.49)
**Education level**		
Degree or above	40,348 (43.79)	39,188 (43.96)
Any other qualification	44,118 (47.88)	42,634(47.83)
No qualification	7673 (8.33)	7319 (8.21)
**Season of accelerometer wear**		
Spring	20,792 (22.57)	20,180(22.64)
Summer	24,068 (26.12)	23,232 (26.06)
Autumn	27,583 (29.94)	26,701 (29.95)
Winter	19,696 (21.38)	19,028 (21.35)
**Smoking status**		
Never	52,952 (57.47)	51,355 (57.61)
Previous	33,382 (36.23)	32,261 (36.19)
Current	5805 (6.30)	5525 (6.20)
**Alcohol consumption**		
Not current	5500 (5.97)	5265 (5.91)
Two or less times a week	42,645 (46.28)	41,153 (46.17)
Three or more times a week	43,994 (47.75)	42,723 (47.93)
**Healthy diet score**	2.69 ± 1.17	2.69 ± 1.17
**Sleep duration**		
< 7 hours/day	31,750 (34.46)	30,659 (34.39)
7-8 hours/day	42,206 (45.81)	40,902 (45.88)
> 8 hours/day	18,183 (19.73)	17,580 (19.72)
**Sleep midpoint (hh:mm)**		
< 02:30	23,823 (25.86)	22,997 (25.80)
02:30-03:30	43,025 (46.70)	41,712 (46.79)
> 03:30	25,291 (27.45)	24,432 (27.41)
**Health status**		
Obesity	17,930 (19.46)	17,194 (19.29)
Diabetes history	4265 (4.63)	4093 (4.59)
Longstanding illness	27,086 (29.40)	26,031 (29.20)
Depression history	8171 (8.87)	7911 (8.87)
Cardiovascular diseases	22,661 (24.59)	21,713 (24.36)
Cancer history	13,428 (14.57)	12,915 (14.49)
**Total MVPA volume (min/week), median [IQR]**	113.83 [158.67]	114.50 [158.83]

Data are mean ± standard deviation or n (%) unless noted otherwise. IQR: interquartile range; MVPA: moderate to vigorous physical activity. To assign categories of timing of MVPA, we divided the clock hours (05:00 to 24:00) into three time windows: morning (05:00-11:00), midday-afternoon (11:00-17:00), and evening (17:00-24:00). These time windows were identified from the exploratory analyses on the timing effects of PA on mortality outcomes (Supplementary Fig. 2). If ⩾ 50% of MVPA occurred during the same time window, participants were assigned into the corresponding timing group. The 50% method we used to define timing of MVPA is similar to what has been previously used [Qian et al. Diabetes Care. 2021]
