# Supplementary material for: Associations of timing of physical activity with all-cause and cause-specific mortality in a prospective cohort study
Source: Nat Commun. 2023 Feb 18;14:930. doi: 10.1038/s41467-023-36546-5 (PMC9938683; doi:10.1038/s41467-023-36546-5)
Supplement: Supplementary file 3 — Description of Additional Supplementary Files [file 41467_2023_36546_MOESM3_ESM.pdf]

Description of Additional Supplementary Files

Title: Supplementary Code

Description: R codes and simulated datasets
