# Supplementary material for: Associations of timing of physical activity with all-cause and cause-specific mortality in a prospective cohort study
Source: Nat Commun. 2023 Feb 18;14:930. doi: 10.1038/s41467-023-36546-5 (PMC9938683; doi:10.1038/s41467-023-36546-5)
Supplement: Supplementary file 5 — Reporting Summary [file 41467_2023_36546_MOESM5_ESM.pdf]

nature portfolio

Corresponding author(s): Jihui ZHANG

Last updated by author(s): Jan 24, 2023

Reporting Summary

Nature Portfolio wishes to improve the reproducibility of the work that we publish. This form provides structure for consistency and transparency in reporting. For further information on Nature Portfolio policies, see our Editorial Policies and the Editorial Policy Checklist.

Statistics

For all statistical analyses, confirm that the following items are present in the figure legend, table legend, main text, or Methods section.

n/a	Confirmed	
☐	☒	The exact sample size (*n*) for each experimental group/condition, given as a discrete number and unit of measurement
☐	☒	A statement on whether measurements were taken from distinct samples or whether the same sample was measured repeatedly
☐	☒	The statistical test(s) used AND whether they are one- or two-sided<br>*Only common tests should be described solely by name; describe more complex techniques in the Methods section.*
☐	☒	A description of all covariates tested
☐	☒	A description of any assumptions or corrections, such as tests of normality and adjustment for multiple comparisons
☐	☒	A full description of the statistical parameters including central tendency (e.g. means) or other basic estimates (e.g. regression coefficient) AND variation (e.g. standard deviation) or associated estimates of uncertainty (e.g. confidence intervals)
☐	☒	For null hypothesis testing, the test statistic (e.g. *F*, *t*, *r*) with confidence intervals, effect sizes, degrees of freedom and *P* value noted<br>*Give P values as exact values whenever suitable.*
☒	☐	For Bayesian analysis, information on the choice of priors and Markov chain Monte Carlo settings
☒	☐	For hierarchical and complex designs, identification of the appropriate level for tests and full reporting of outcomes
☒	☐	Estimates of effect sizes (e.g. Cohen's *d*, Pearson's *r*), indicating how they were calculated

Our web collection on statistics for biologists contains articles on many of the points above.

Software and code

Policy information about availability of computer code

Data collection	All data in this study were derived from the UK Biobank.
Data analysis	R version 4.0.4; R packages: 'mice' (v3.13.0), 'survival' (v3.2-11), 'interactionR' (v0.1.3.9000), and 'cmprsk' (v2.2-10); SPSS V26.

For manuscripts utilizing custom algorithms or software that are central to the research but not yet described in published literature, software must be made available to editors and reviewers. We strongly encourage code deposition in a community repository (e.g. GitHub). See the Nature Portfolio guidelines for submitting code & software for further information.

Data

Policy information about availability of data

All manuscripts must include a data availability statement. This statement should provide the following information, where applicable:

- Accession codes, unique identifiers, or web links for publicly available datasets
- A description of any restrictions on data availability
- For clinical datasets or third party data, please ensure that the statement adheres to our policy

The UK Biobank data are available from the UK Biobank and can be accessed by researchers on application (www.ukbiobank.ac.uk/). Source data are provided with this paper.

Human research participants

Policy information about studies involving human research participants and Sex and Gender in Research.

Reporting on sex and gender	The sex information is acquired from central registry at recruitment, but in some cases updated by the participant. Hence it may contain a mixture of the sex the National Health Service (NHS) had recorded for the participant and self-reported sex. Taking morning group as a reference, both the midday-afternoon and mixed timing groups, but not evening group, showed significantly decreased risks of all-cause and CVD mortality. We also conducted multiplicative and additive interaction analyses, joint associations, and subgroup analyses on sex. We found that the observed protective effects of the midday-afternoon and mixed timing groups seemed to be stronger among males than females.
Population characteristics	92,139 UK Biobank participants (mean age, 62 years; 56% female) with valid accelerometer data were recruited. The baseline characteristics of 92,139 participants are shown in Table 1. Overall, four timing groups presented similar profiles for sociodemographic, lifestyle, and health status. However, mixed group showed a lower Townsend deprivation index than other groups. The morning group had a lower education level than other groups. The evening and mixed groups tended to have shorter sleep duration, since they were more from the group of <7 hours/day than morning and midday-afternoon groups. The morning group were more from the group of sleep midpoint earlier than 02:30 than other groups. Additionally, we found that morning and evening groups had fewer moderate-to-vigorous intensity physical activity minutes than other two groups.
Recruitment	The UK Biobank is a large population-based prospective cohort that recruited approximately 500,000 participants aged 40-73 years in the United Kingdom. Participants visited one of 22 assessment centers across England, Scotland, and Wales. 236,519 UK Biobank participants were invited to participate in an accelerometer study. A total of 103,712 raw accelerometer datasets were received for data analysis. Participants who accepted accelerometry measurement showed similar baseline demographic and health-related characteristics as those who declined the measurement (Khurshid et al. European Heart Journal. 2021).<br>Raw accelerometer data from 103,682 participants were further processed by the UK Biobank accelerometer expert working group. In addition, according to the exclusion criteria, 11,543 participants were excluded, and 92,139 participants (88.87%) with valid data were finally included in the current study. We conducted multiple imputations to assign any missing covariate values. Overall sample and complete case sample showed similar baseline characteristics (supplementary table 3).
Ethics oversight	UK Biobank has approval from the North West Multi-centre Research Ethics Committee (MREC) as a Research Tissue Bank (RTB) approval. This approval means that researchers do not require separate ethical clearance and can operate under the RTB approval. Informed consents were obtained from all participants. The ethical approval for the UK Biobank is extensively detailed and described online: https://www.ukbiobank.ac.uk/learn-more-about-uk-biobank/about-us/ethics. This current study was undertaken under project 58082.

Note that full information on the approval of the study protocol must also be provided in the manuscript.

Field-specific reporting

Please select the one below that is the best fit for your research. If you are not sure, read the appropriate sections before making your selection.

- [x] Life sciences
- [ ] Behavioural & social sciences
- [ ] Ecological, evolutionary & environmental sciences

For a reference copy of the document with all sections, see nature.com/documents/nr-reporting-summary-flat.pdf

Life sciences study design

All studies must disclose on these points even when the disclosure is negative.

Sample size	This is a cohort study that assesses the exposure-outcome associations. in this case, the sample size is determined by the number of events required to perform multivariable regression models. According to the rule-of-thumb estimation, at least ten events are required per variable (including dummy variables) in the model (Riley et al. BMJ. 2020). In the fully adjusted models, we included a total of 24 variables (including dummy variables) in the Cox regression models. Thus, at least 240 events for each outcome were required. The events for all-cause mortality, cardiovascular mortality, and cancer mortality were 3088, 1076, and 1872, respectively. Therefore, the sample size of this study should be sufficient.
Data exclusions	The data exclusion criteria were pre-established and documented in Methods and Supplementary Methods. The exclusion criteria are as follows: 1) those who withdrew from UK Biobank; 2) those who had no physical activity data in any one hour of the 24-hour cycle; 3) Similar to the previous study, those who had high nocturnal activity (>10% physical activity accumulated between 01:00 and 04:00), as we focused on individuals with a diurnal lifestyle; 4) those with unreliable or invalid accelerometry data: i) unexpectedly small or large size (UK Biobank Field ID: 90002); ii) less than 72 h or did not provide data for all 1-h periods within a 24-h cycle during the 7-day data collection (Field ID: 90015); iii) not well-calibrated (Field ID: 90016); iv) recalibrated using the previous accelerometer record from the same device worn by a different participant (Field ID: 90017); v) data with a non-zero count of interrupted recording periods (Field ID: 90180); vi) data with more than 768 (Q3 + 1.5 × IQR) data recording errors (Field ID: 90182). In total 11,543 participants were excluded. Finally, 92,139 participants (88.87%) with valid data were included in the current study.
Replication	This is a population-based cohort study, and we have not yet replicated the findings in other samples. However, we used different statistical

	methods to verify our findings: 1) carefully controlling for a wide range of potential covariates; 2) running analyses on the associations between continuous variables regarding timing groups and outcomes; 3) comprehensive sensitivity analyses [i.e., using different cutoffs for defining timing groups, using competing risk regression models, using the dataset without imputation, excluding participants with shift work history, excluding participants who wore accelerometers during the daylight saving time transition, additionally adjusting for health-related variables potentially on the causal pathway, excluding events within the first year of follow-up, censoring up to Dec 31, 2019 (the start of COVID-19 pandemic in the UK), and using the subsample with ≥ 6 days of accelerometer wear]; and 4) validated in multiple testing correction by the Benjamini-Hochberg False Discovery Rate method. Our main findings were rather robust and consistent across these analyses.
Randomization	This is an observational cohort study, and randomization is not applicable in this study. However, we carefully controlled for a wide range of potential covariates in the Cox regression models to mimic randomization.
Blinding	The investigators were blinded to the exposure group during the statistical analyses for testing the exposure-outcome associations.

Reporting for specific materials, systems and methods

We require information from authors about some types of materials, experimental systems and methods used in many studies. Here, indicate whether each material, system or method listed is relevant to your study. If you are not sure if a list item applies to your research, read the appropriate section before selecting a response.

Materials & experimental systems

n/a	Involved in the study
☒	☐ Antibodies
☒	☐ Eukaryotic cell lines
☒	☐ Palaeontology and archaeology
☒	☐ Animals and other organisms
☒	☐ Clinical data
☒	☐ Dual use research of concern

Methods

n/a	Involved in the study
☒	☐ ChIP-seq
☒	☐ Flow cytometry
☒	☐ MRI-based neuroimaging
